# Prevalence of Selected Non-Metric Dental Traits in Indo-Nepalese and Tibeto-Nepalese Ethnic Groups of Western Hilly Region

**DOI:** 10.31729/jnma.4603

**Published:** 2019-10-31

**Authors:** Sanjay Kumar Sah, Santosh Kandel, Raju Shrestha, Alok Atreya

**Affiliations:** 1 Department of Forensic Medicine and Toxicology, Lumbini Medical College and Teaching Hospital, Palpa, Nepal; 2 Department of Dentistry, Lumbini Medical College and Teaching Hospital, Palpa, Nepal

**Keywords:** *cusp of carabelli*, *ethnic groups*, *identification*, *incisor*

## Abstract

**Introduction:**

Additional anatomic features present on teeth are called non-metric dental traits. Carabelli’s cusp and shovelling are such traits which are mostly evaluated for identification of ethnicity. The present study aims to determine the prevalence of Carabelli’s cusp and shovelling among Indo-Nepalese and Tibeto-Nepalese ethnic group.

**Methods:**

A descriptive cross-sectional study was conducted in a tertiary hospital from March 22 to June 22 2019 after obtaining ethical approval from the institutional review committee. The study was conducted among 274 patients and convenient sampling method was applied. Data were analyzed by the help of Statistical Package for Social Sciences version 21.

**Results:**

Among 274 cases, 153 (55.84%) cases were Indo-Nepalese and 121 (44.16%) were Tibeto-Nepalese. Carabelli’s cusp (16/26) was noticed in 87 (56.86%) of Indo-Nepalese and 45 (37.19%) of Tibeto-Nepalese. Shovelling (11, 12, 21, 22) was present in 47 (30.71%) Indo-Nepalese and 79 (65.28%) of Tibeto-Nepalese. Further, Carabelli’s cusp (16/26) was found in 77 (53.10%) females and 55 (55.12%) males. Shovelling was present in 75 (51.72%) females and 51 (39.53%) males. Bilateralism with respect to Carabelli’s cusp was present in 82 (62.2%) cases. Presence of bilateral shovelling on upper central incisors and lateral central incisors were among 117 (94.35%) and 56 (91.80%) respectively.

**Conclusions:**

Carabelli’s cusps are frequently found in Indo-Nepalese ethnic group and shovelling of teeth most commonly present in Tibeto-Nepalese population. Further, bilaterism for shovelling of teeth is more common than bilaterism for cusp of Carabelli.

## INTRODUCTION

Identification of a person based on gender and origin can be successfully carried out by evaluation of additional anatomic features present on the teeth known as non-metric dental traits. Due to their stability and capacity to withstand adverse environment, these traits have been widely applied as a study purpose in forensic anthropology.^1-2^ Cusp of Carabelli and shovelling are two major traits which are frequently observed in human dentition.^3^ cusp of Carabelli, first described in the year 1842 by Carabelli, is an additional lobe of varying degree present on molar teeth, primarily on the first molar.^4^ Shovel incisors are concave and gouged lingual surface due to the presence of marginal ridge.^5^ Indo-Nepalese and Tibeto-Nepalese are two major ethnic groups in residing western hilly region of Nepal.^6^

The present study aims to determine the prevalence of dental morphological traits in both the ethnic groups of western hilly region of Nepal.

## METHODS

The descriptive cross sectional study was carried out in the Department of Dentistry, Lumbini Medical College Teaching Hospital Palpa, Nepal from March 22 to June 22 2019. The study was carried out after obtaining ethical approval from the Institutional Review Committee (IRC-LMC 20A/019). Sample size was calculated by applying formula:

n= Z^2^ × p × q/e^2^

where,

n= Sample size
Z=1.645 at Confidence Interval, 90%
p= prevalence in previous similar studies (63%)^7^
e= margin of error= 5%

Calculated sample size was 252 and then the study was conducted in 274 patients (145 females and 129 males) of age 14 years and above attending OPD in dental department at Lumbini Medical College Teaching Hospital, Palpa. Convenient sampling method was applied.

Patients with fully erupted permanent first and second molars and all upper permanent incisors were included and the patients having history of cross ethnicity, having restored, carious and missing first and second molars, upper incisors of any side were excluded from the study.

An informed consent was taken prior to examination. Ethnicity was determined based on history of inhabitant, community, homeland and language. The palatal surface of mesio-palatal cusp of left and right first upper molars (16, 26) of the subjects were evaluated for cusp of Carabelli. The shovel trait was evaluated in upper incisors (11, 21, 12, 22). Teeth were examined using mouth mirror and dental probe.

Data were analyzed by the help of Statistical Programme for Social Sciences (SPSS) version 21.

## RESULTS

Presence of Carabelli’s cusp on either of the upper first molar in 6fg Indo-Nepalese ethnic group was 87 (56.86%) in numbers while in 66 (43.14%) cases Carabelli’s cusp was absent. Among Tibeto-Nepalese ethnic group, Carabelli’s cusp was found on 45 (37.19%) patients and absent in 76 (62.81%) patients. Likewise, the numbers of Indo-Nepalese population in whom shovelling was present on either of upper central and lateral incisors was 47 (30.71%) and in 106 (69.29%) it was absent. Similarly, in Tibeto-Nepalese ethnic group shovelling was present in 79 (65.28%) of cases while in 42 (34.72%) cases shovelling was absent. The cusp of Carabelli’s was more frequently found on Indo-Nepalese population in comparison to the Tibeto-Nepalese population. Likewise, shovelling was significantly high on Tibeto-Nepalese populations (Table 1).

**Table 1:** Frequency of Carabelli’s cusp and shovelling in Indo-Nepalese and Tibeto-Nepalese.

Ethnicity	Cusp of Carabelli (16, 26)		Shovelling (22,21, 12,11)	
Indo-	Present	Absent	Present	Absent
Nepalese	n (%)	n (%)	n (%)	n (%)
	87	45	47	106
	(56.86)	(43.14)	(30.71)	(69.29)
Tibeto-	45	76	79	42
Nepalese	(37.19)	(62.81)	(65.28)	(34.72)

Carabelli’s cusp on either of upper first molar was found in 77 (53.10%) females and 55 (55.12%) males while absent in 68 (46.89%) females and 74 (44.88%) males. Shovelling on either of upper central and lateral incisors was observed in 75 (51.72%) females and 51 (39.53%) males.

Based on ethnicity, Carabelli’s cusp was present in 50 (64.93%) Indo-Nepalese females and 27 (35.06%) Tibeto-Nepalese females. Likewise, 37 (67.27%) Indo-Nepalese males have cusp of Carabelli on either of first molar and 18 (32.77%) Tibeto-Nepalese have the same. Shovelling was present in 45 (60%) of Tibeto-Nepalese females and 30 (40%) of Indo-Nepalese females. In case of males, 34 (66.66%) of Tibeto-Nepalese and 17 (33.34%) Indo-Nepalese have shovelling on upper incisors (Table 2).

**Table 2:** Sex-wise distribution of Carabelli’s cusp and shovelling.

	Cusp of Carabelli(26,16)		Shovelling(22,21,12,11)	
Sex	Present n (%)	Absent n (%)	Present n (%)	Absent n)
Female	77 [IN50+TN27] (53.10)	68 (46.89)	75 [IN30+TN45] (51.72)	70 (48.28)
Male	55 [IN37+TN18] (55.12)	74 (44.88)	51 [IN17+TN34] (39.53)	78 (60.47)

*IN=Indo-Nepalese

*TN=Tibeto-Nepalese

Bilateral presence of Carabelli’s cusp was observed in 82 (62.12%) cases while in 50 (37.88%) cases there was unilateral presence of Carabelli’s cusp. Likewise, presence of bilateral shovelling on upper central incisor was observed in 117 (94.35%) and in 7 (5.65%) cases there was unilaterism of shovelling. For upper lateral incisor, bilaterism was found on 56 (91.80%) cases and unilaterism was found on 5 (8.2%) cases (Table 3).

**Table 3:** Bilateralism of Carabelli’s cusp and shovelling.

Traits	Bilateralism Present n (%)	Absent n (%)	Total n (%)
Carabelli’s cusp(16/26)	82 (62.12)	50 (37.88)	132 (100)
Shovelling (11/21)	117 (94.35)	7 (5.65)	124 (100)
Shovelling (12/22)	56 (91.80)	5 (8.20)	61 (100)

## DISCUSSION

Evaluation of non-metric dental traits can be applied for identification of ethnic group as there is specific prevalence of these traits among particular ethnic group. Nepal is one of the countries having inhabitants of multi-ethnic population. Among them most of the population living in western part of hilly region fall in Indo-Nepalese ethnicity and Tibeto-Nepalese ethnicity. Indo-Nepalese ethnic group comprises of population living at more fertile lower hills, river valleys and Terai plains adjoining boundary of India. The Tibeto-Nepalese ethnic group represents the community occupying the higher hills from west to east. There are numerous studies conducted to differentiate the Mongoloid and Caucasian ethnicity based on presence of cusp of Carabelli and shovel shaped incisors, but only few works have been done previously among the Nepalese population.^8-9^ This study aims to find out prevalence of Carabelli’s cusp and shovelling among the population of above mentioned group residing in western hilly region.

In this study, among 274 individuals Carabelli’s cusp on upper first molar was observed in 48.2% and shovelling on upper incisors was found on 46% individuals. Study done by M kirtigha et al. on Indian population reported 40.5% of subjects had cusp of Carabelli on first molar and 68.2% had shovelling on upper central incisors.^10^ Sadatullah S et al. observed Carabelli’s cusp on 41.7% of Saudi Population.^11^ A study conducted on Nepalese population by Subedi et al. showed presence of Carabelli trait on 68.3%.^7^

Based on ethnicity, cusp of Carabelli was found on 56.86% of Indo-Nepalese when compared to the Tibeto-Nepalese where it was present on 37.19%. Similarly, shovelling in upper incisors was found on 65.28% of Tibeto-Nepalese population and in 30.71% of Indo-Nepalese population. So, this study reveals high percentage of Carabelli’s cusp in Indo-Nepalese population and more prevalence of shovelling on Tibeto-Nepalese population. This study is consistent with the study where presence of shovelling was higher in Mongolids.^8^ Study done by Kharat DU et al. showed shovelling range of 20-25% in population of Sudan and Egypt.^12^ A study by Srivastava et al. showed only 8% Tamil population having shovelling.^13^

Overall sex wise distribution of cusp of Carabelli and shovelling shows no significant sexual dimorphism. This finding is similar to study conducted by Talabani R et al. in Iraq.^14^ Subedi et al. presented similar observation on Nepalese population.^7^ But this study showed Indo-Nepalese ethnic females have higher prevalence of Carabelli’s cusp and Tibeto-Nepalese females have higher prevalence of shovelling with comparison to their male counterparts.

In present study bilateral distribution of Carabelli’s cusp in upper first molar is 62.12%. Subedi et al. reported 73.7% in Nepalese population.^7^ A Nigerian study showed bilaterism of 70.71% with respect to upper first molar.^15^ Shethri SA reported it to be 82.2% in Saudi population.^16^ Our findings shows 94.35% bilaterism of shovelling with respect to upper central incisor and 91.80% with respect to lateral central incisors suggesting shovelling has higher prevalence of shovelling than cusp of Carabelli in Nepalese population.

In this study only the presence of selected non-metric dental traits namely Carabelli’s cusp and shovelling were observed and again those observations were limited to selected teeth. For Carabelli’s cusp only upper first molars were evaluated and for shovelling only upper incisors were evaluated based on frequency of their presence. So, further studies among various ethnic groups with respect to various non-metric traits are necessary for validation of use of non-metric dental traits in determining the ethnic identification. Results from our studies can be applied to the population around Palpa district region.

## CONCLUSIONS

Carabelli’s cusps are frequently found in Indo-Nepalese ethnic group and shovelling of teeth most commonly present in Tibeto-Nepalese population. Further, bilaterism for shovelling of teeth is more common than bilaterism for cusp of Carabelli.

## Conflict of Interest:

None.
